# Coupling between global brain blood oxygen level‐dependent activity and cerebrospinal fluid dynamics in young endurance athletes

**DOI:** 10.1113/EP093289

**Published:** 2026-02-26

**Authors:** Daisuke Hoshi, Marina Fukuie, Ryota Asahara, Tsubasa Tomoto, David C. Zhu, Rong Zhang, Keigo Ohyama‐Byun, Seiji Maeda, Jun Sugawara, Takashi Tarumi

**Affiliations:** ^1^ Human Informatics and Interaction Research Institute National Institute of Advanced Industrial Science and Technology Tsukuba Japan; ^2^ Japan Society for the Promotion of Science Tokyo Japan; ^3^ Department of Sports Sciences Japan Institute of Sports Sciences Tokyo Japan; ^4^ Integrated Research Center for Self‐Care Technology National Institute of Advanced Industrial Science and Technology Tsukuba Japan; ^5^ Health and Medical Research Institute National Institute of Advanced Industrial Science and Technology Tsukuba Japan; ^6^ Institute for Exercise and Environmental Medicine Texas Health Presbyterian Hospital Dallas Dallas Texas USA; ^7^ Department of Radiology and Gruss Magnetic Resonance Research Center Albert Einstein College of Medicine Bronx New York USA; ^8^ Department of Neurology University of Texas Southwestern Medical Center Dallas Texas USA; ^9^ Department of Internal Medicine University of Texas Southwestern Medical Center Dallas Texas USA; ^10^ Institute of Health and Sports Sciences University of Tsukuba Tsukuba Japan; ^11^ Sports Physiology, School of Sport Sciences Waseda University Saitama Japan

**Keywords:** brain waste clearance, cerebral haemodynamics, cerebrospinal fluid, endurance training, glymphatic system, resting‐state functional magnetic resonance imaging

## Abstract

Cerebrospinal fluid (CSF) contributes to brain waste clearance through its coupling with cerebral haemodynamics. Aerobic exercise promotes brain health, but its influence on brain waste clearance remains unclear. This study examined the coupling between CSF and cerebral haemodynamics in endurance athletes. Fifteen young male endurance athletes were compared with 16 age‐matched sedentary male controls. Resting‐state functional magnetic resonance imaging was used to obtain brain global blood oxygen level‐dependent (gBOLD) and CSF inflow signals. The gBOLD–CSF coupling was estimated using cross‐correlation function and transfer function analyses in time and frequency domains, respectively. The magnitude of gBOLD and CSF oscillations was also quantified by power spectral density. The cross‐correlation‐derived gBOLD–CSF coupling was similar between athletes and sedentary controls (−0.30 ± 0.26 vs. −0.33 ± 0.22, *P* = 0.753). Power spectral density of the CSF inflow signal was significantly greater in athletes (2.83 [2.19, 3.47] *z* ^2^/Hz) than sedentary individuals (1.84 [1.22, 2.46] *z* ^2^/Hz, *P* = 0.030), whereas that of the gBOLD signal did not differ between groups. Although transfer function gain did not show a statistically significant group difference, a moderate effect size was observed (1.50 [–0.36, 3.36] vs. 1.24 [−0.06, 2.55], *P* = 0.051, effect size: 0.523). Transfer function phase and coherence were comparable between groups. Significant frequency dependence was observed for gBOLD power spectral density as well as transfer function gain, phase and coherence across groups. Endurance athletes demonstrated greater CSF fluctuation compared with sedentary individuals. Our findings suggest that endurance training may be linked to changes in CSF dynamics.

## Abstract (highlights)

- **What is the central question of this study?**
  Regular aerobic exercise benefits brain health, but the mechanisms remains unclear. Does coupling between global blood‐oxygen‐level‐dependent (gBOLD) activity and cerebrospinal fluid (CSF) dynamics, a process thought to support brain waste clearance, contribute to this effect?
- **What is the main finding and its importance?**
  Endurance athletes showed a larger amplitude of CSF fluctuation than sedentary individuals. Transfer function gain between gBOLD and CSF did not show a statistically significant group difference but demonstrated moderate effect size (Cohen's *d* = 0.523). These findings suggest that aerobic exercise training may be associated with changes in CSF dynamics.

## 1 INTRODUCTION

The brain has one of the highest metabolic rates in the human body despite its limited energy storage. Consequently, cerebral circulation must provide a continuous supply of arterial blood and simultaneously remove metabolic byproducts to maintain microenvironmental homeostasis (Hedden et al., 2013; Nedergaard & Goldman, 2020; Raichle & Gusnard, 2002; Xing et al., 2017). Cerebrospinal fluid (CSF) has been shown to contribute to glymphatic‐mediated clearance of neurotoxic substances from the brain (Benveniste et al., 2019; Boland et al., 2018; Kipnis et al., 2025). CSF flows from the perivascular space into the interstitial space where it mixes with interstitial fluid and facilitates the removal of metabolites into the perivenous space (Rasmussen et al., 2022; Tarasoff‐Conway et al., 2016). The autonomic nervous system may play an important role in this process as sleep, a state characterized by parasympathetic dominance, appears to promote glymphatic function partly by enhancing CSF pulsatility (Bolt et al., 2025; Fultz et al., 2019; Rasmussen et al., 2022).

Regular aerobic exercise is associated with beneficial effects on brain health (Tarumi, Tomoto et al., 2025), but its impact on brain waste clearance remains less understood. Animal studies suggest that 5‐ to 6‐week voluntary exercise can promote expression and polarization of astrocytic aquaporin‐4 water channels, thereby supporting glymphatic transport (He et al., 2017; von Holstein‐Rathlou et al., 2018). In humans, a 12‐week cycling intervention was reported to increase putative glymphatic and meningeal lymphatic vessel flow measured by contrast‐enhanced magnetic resonance imaging (MRI) (Yoo et al., 2025). These findings indicate a potential link between habitual physical activity and enhanced brain waste clearance. In contrast, CSF flow has been observed to decrease during exercise despite increased brain activity and metabolic demand (Tarumi, Yamabe, Fukuie, Zhu et al., 2021). This raises the possibility that glymphatic activity may be suppressed acutely during exercise and preferentially reactivated during recovery or sleep. Endurance athletes who routinely engage in high‐intensity aerobic training often exhibit heightened parasympathetic activity at rest (Aubert et al., 2003). Such autonomic nervous adaptations could potentially enhance the efficiency of brain waste clearance even during wakefulness; however, this hypothesis remains to be tested.

CSF dynamics are closely coupled with global cerebral haemodynamics measured by resting‐state functional MRI (Fultz et al., 2019; Zhu et al., 2015). Reduced strength of this coupling has been associated with toxic protein accumulation in patients with Alzheimer's disease (Han et al., 2023; Han, Lee et al., 2024). Global blood oxygen level‐dependent (gBOLD) signals below 0.1 Hz have recently been recognized as structured, brain‐wide neurovascular activity that reflects an acute arousal state (Gu et al., 2021; Liu et al., 2021) and is modulated by autonomic nervous activity (Bolt et al., 2025). Fluctuations in gBOLD signals have been linked to vasomotor dynamics (Kiviniemi et al., 2016) and may relate to perivascular fluid movement (Mestre et al., 2018), and have also been associated with astroglial calcium waves (Wang et al., 2018) as well as CSF dynamics (Fultz et al., 2019). Collectively, these observations suggest that gBOLD–CSF coupling may provide a non‐invasive surrogate marker of glymphatic‐mediated brain clearance efficiency.

To date, most studies assessing gBOLD–CSF coupling have relied on cross‐correlation function analysis applied to bandpassed gBOLD and CSF time‐series data at 0.01–0.1 Hz. Vasomotor waves within this frequency range are influenced by both sympathetic and parasympathetic activity, while slow pressure fluctuations from vascular smooth muscle contractions may also affect glymphatic‐related CSF flow (Kiviniemi et al., 2016). These observations highlight the importance of frequency‐dependent features of gBOLD and CSF signals. However, because cross‐correlation analysis relies on simple Pearson correlation, it is limited to describing the dynamic relationship between the two signals and does not allow identification of the underlying contributors to each signal. By applying frequency‐domain analyses, it is possible to characterize the frequency components of each signal and to evaluate, in greater detail, the power of signal fluctuations as well as frequency‐specific magnitude (gain), temporal (phase), and coherence relations between gBOLD and CSF signals.

Building on these methodological considerations, the present study examined gBOLD–CSF coupling in male endurance athletes and age‐matched sedentary controls using both cross‐correlation function and transfer function analyses. We hypothesized that endurance athletes would exhibit stronger gBOLD–CSF coupling compared with sedentary individuals and that fluctuations of gBOLD and CSF signals would be greater in athletes. We also hypothesized that frequency‐dependent differences in the gBOLD and CSF dynamics would be observed, as revealed by spectral and transfer function metrics.

## 2 METHODS

### 2.1 Participants

Fifteen young male endurance athletes and 16 age‐matched sedentary male adults participated in this study. Athletes were recruited from the University of Tsukuba track‐and‐field team and were actively training for middle‐ and long‐distance events. Their training typically included ∼12 sessions per week (∼60 min each): four high‐intensity interval runs, four moderate‐intensity continuous runs, and four low‐intensity jogging sessions. In addition, they performed strength training (e.g., bench press, squat, deadlift, snatch) twice per week for ∼30 min per session. Sedentary participants had not engaged in structured exercise for at least 3 years.

Exclusion criteria for both groups included a history of cardiovascular, cerebrovascular, renal or neurological disorders; cigarette smoking; use of medications affecting cardiovascular function (e.g., antihypertensives, antilipidemics, antidiabetics); claustrophobia; or the presence of MRI‐incompatible metal implants. The study protocol was approved by the Institutional Review Board of the National Institute of Advanced Industrial Science and Technology (Hito 2018–873) and was conducted in accordance with the *Declaration of Helsinki* and the Belmont Report. Written informed consent was obtained from all participants.

Some data from this cohort have been reported previously in studies examining proximal aortic compliance (Tarumi, Yamabe, Fukuie, Kimura et al., 2021), cerebral white matter microstructure (Tarumi et al., 2022), and aortic input impedance (Fukuie et al., 2024). The present study, however, addresses a different objective by focusing on gBOLD–CSF coupling to explore its potential relevance for brain waste clearance. Due to missing fMRI data in some participants (three sedentary individuals), the number of participants included here differs from those prior reports (Fukuie et al., 2024; Tarumi et al., 2022; Tarumi, Yamabe, Fukuie, Kimura et al., 2021).

### 2.2 Study protocol

Participants were instructed to fast for at least 2 h and to abstain from caffeine, alcohol and strenuous exercise for at least 24 h prior to testing. Upon arrival, participants completed medical history, physical activity and MRI safety questionnaires. Height and body mass were measured to calculate body mass index (BMI). After 10 min of seated rest, heart rate and brachial blood pressure were measured at least twice using an automated monitor (HEM‐7130; Omron Corporation, Kyoto, Japan). An MRI scan was then performed in the supine position using a 3‐tesla scanner (Ingenia, Philips Healthcare, Best, Netherlands) with a 32‐channel head coil (dStream). During the resting‐state functional MRI acquisition, participants were instructed to fixate on a cross displayed on the monitor. Wakefulness was monitored by the experimenter using an in‐bore camera throughout the scan and by communicating with participants between the scan protocols. MRI measurements were conducted at a mean time of 11.47 h ± 2 h 31 min (athlete 11.57 h ± 2 h 25 min, sedentary 11.39 h ± 2 h 35 min). Maximal oxygen uptake (${\dot{V}}_{O_{2}\max}$) was measured in athletes on a separate day.

### 2.3 MRI data acquisition

Following survey imaging, T1‐weighted 3D magnetization‐prepared rapid acquisition with gradient echo (MPRAGE), resting‐state functional MRI, and T2‐weighted fluid‐attenuated inversion recovery (FLAIR) images were collected. MPRAGE data were acquired with the following parameters (Tarumi et al., 2022; Zhu et al., 2015): voxel resolution = 1 × 1 × 1 mm^3^, echo time (TE) = 3 ms, repetition time (TR) = 7 ms, flip angle = 8°, sensitivity encoding (SENSE) factor = 2.2, field of view (FOV) = 256 × 256 mm^2^ (non‐oblique), sagittal slices = 176 (no gap), and total scan duration = 5 min 37.9 s. Resting‐state functional MRI data were acquired using an echo‐planar imaging (EPI) sequence with the following parameters (Zhu et al., 2015): TE = 28 ms, TR = 2500 ms, flip angle = 80°, in‐plane resolution = 3.44 × 3.34 mm^2^, matrix = 64 × 66, FOV = 220 × 220 mm^2^ (non‐oblique), axial slices = 48 (no gap), slice thickness = 3 mm, 253 scans (including 3 dummy scans), and total scan duration = 10 min 32.5 s. The resting‐state functional MRI acquisition covered the whole brain, with the lower edge placed at the bottom of the cerebellum to optimize CSF signal detection alongside gBOLD signals. To improve brain segmentation and volume calculation, FLAIR images were acquired with the following parameters (Fischl, 2012; Tarumi, Won et al., 2025): in‐plane resolution = 1 × 1 mm^2^, TE = 125 ms, TR = 11,000 ms, inversion time = 2800 ms, FOV = 240 × 240 mm (non‐oblique), axial slices = 48 (no gap), slice thickness = 3 mm, and total scan duration = 6 min 36 s.

### 2.4 Image preprocessing and signal extraction

**FIGURE 1:**
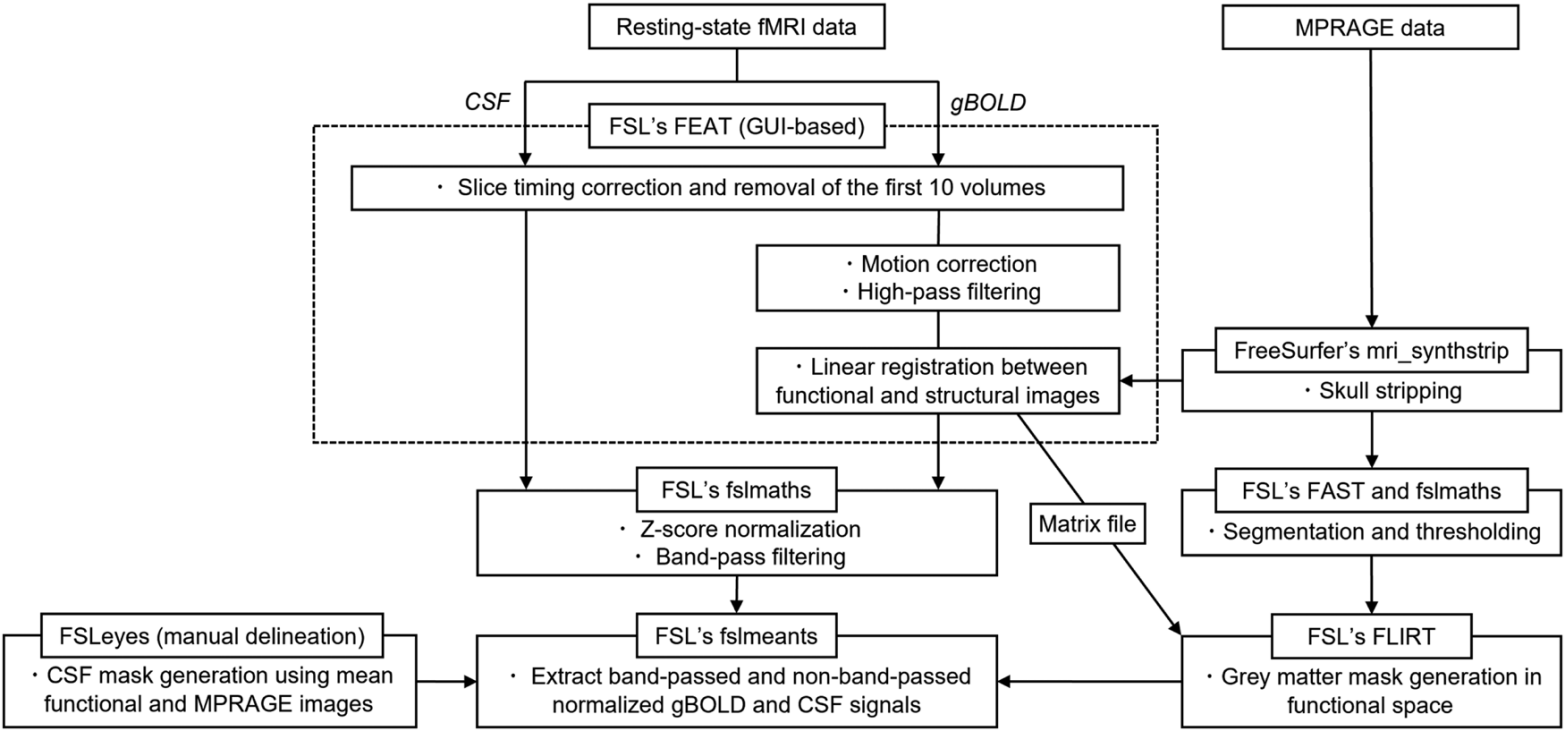
Flow chart of preprocessing steps for resting‐state functional magnetic resonance imaging and T1‐weighted magnetization‐prepared rapid acquisition with gradient echo (MPRAGE) data. CSF, cerebrospinal fluid; fMRI, functional magnetic resonance imaging; gBOLD, global blood oxygen level‐dependent; MPRAGE, T1‐weighted 3D magnetization‐prepared rapid acquisition with gradient echo.

Figure 1 and Appendix Table A1 summarize the preprocessing pipeline and codes used for gBOLD and CSF signal extraction. Image preprocessing was performed on a computer running macOS (version 12.7.6) using the FMRIB Software Library (FSL; version 6.0.7.15) and FreeSurfer (version 7.4.1), following previously published methods (Fultz et al., 2019; Han et al., 2021).

High‐resolution T1‐weighted MPRAGE images were skull‐stripped using FreeSurfer's *mri_synthstrip* command with the *–no‐csf* option, which excludes sulcal and ventricular cerebrospinal fluid (CSF) and produces a tighter brain mask (Hoopes et al., 2022). The skull‐stripped brain structural image was segmented into grey matter, white matter and CSF partial volume estimate maps using FSL's Automated Segmentation Tool (*FAST*) (Zhang et al., 2001). A binary grey matter mask was created by thresholding the grey matter partial volume map at a probability of 0.5. This grey matter mask was registered to functional space using FSL's Linear Image Registration Tool (*FLIRT*), applying the high‐resolution‐to‐functional transformation matrix generated by FSL's FMRI Expert Analysis Tool (*FEAT*) and using nearest‐neighbour interpolation to preserve mask integrity (Jenkinson et al., 2002; Smith et al., 2004). MPRAGE images were also used to compute total brain volume using FreeSurfer's *recon‐all* pipeline with FLAIR images included to improve pial surface delineation (Fischl, 2012). Cortical reconstruction and volumetric segmentation were based on the Desikan–Killiany atlas (Desikan et al., 2006).

**FIGURE 2:**
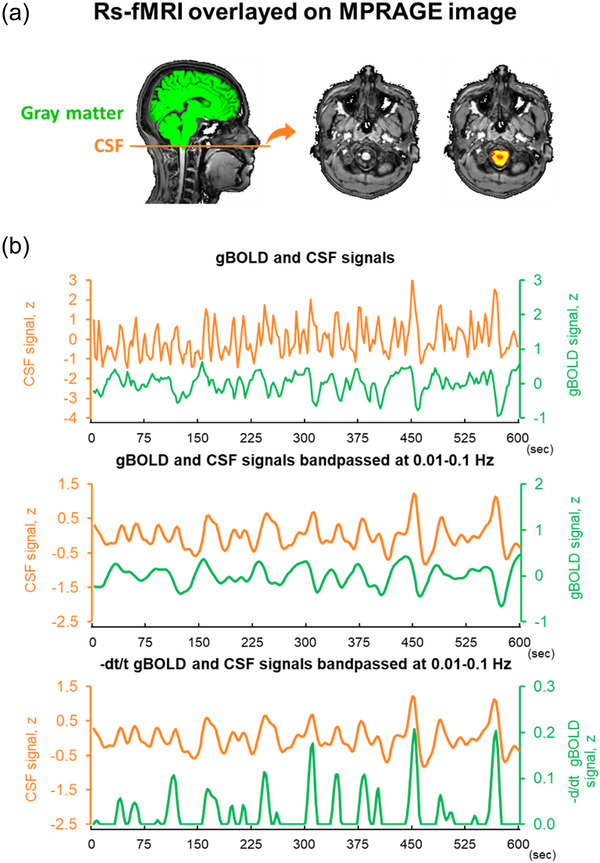
(a) Global blood oxygen level‐dependent (gBOLD) signal was extracted while averaging within a grey matter mask (green marker). Cerebrospinal fluid (CSF) inflow signal was extracted from the bottom slice of the functional image (orange line) to maximize sensitivity to CSF inflow effects. For each participant, a CSF mask was manually drawn on the mean functional image confirmed with T1‐weighted magnetization‐prepared rapid acquisition with gradient echo (MPRAGE) image, and voxels with high‐intensity CSF inflow signals were delineated. (b) Normalized, non‐bandpassed gBOLD and CSF inflow signals were used for frequency‐domain analyses (top), whereas bandpassed signals at 0.01–0.1 Hz were used for cross‐correlation function analysis (middle). Following previous studies (Fultz et al., 2019), the time derivative of gBOLD signal was computed by multiplying by –1 and setting negative values to zero (bottom).

Resting‐state functional MRI data were preprocessed using FSL's FEAT (Smith et al., 2004). Preprocessing steps included slice‐timing correction, removal of the first 10 volumes to ensure magnetization equilibrium, motion correction, brain extraction, and temporal high‐pass filtering using FEAT's default settings. The preprocessed functional data (*filtered_func_data*) were used for subsequent analyses. To normalize signal amplitude across voxels, voxelwise temporal *z*‐score normalization was performed by subtracting the temporal mean and dividing by the temporal standard deviation of each voxel time series using *fslmaths*. In addition, the data were bandpass‐filtered using FSL's temporal filtering function (*fslmaths ‐bptf*) with cutoffs corresponding to approximately 0.007–0.1 Hz (TR = 2.5 s), consistent with frequency ranges commonly examined in resting‐state fMRI studies. Using FSL's *fslmeants*, the normalized gBOLD time‐series data were extracted from both bandpass‐filtered and non‐bandpass‐filtered datasets using the grey matter mask in functional space (Figure 2b).

CSF signal extraction was performed using a preprocessing approach distinct from the gBOLD pipeline. Slice‐timing correction and removal of the first 10 volumes were applied to the functional data; however, motion correction and temporal high‐pass filtering were intentionally omitted to preserve slice‐specific voxel position and low‐frequency temporal information critical for detecting CSF inflow–related signal changes (Fultz et al., 2019). Accordingly, CSF preprocessing was conducted separately from the standard FEAT pipeline used for gBOLD processing. CSF signals were extracted from the most inferior slice of the functional image volume, corresponding to the cerebellar base, to maximize sensitivity to CSF inflow dynamics. A CSF region‐of‐interest mask was manually delineated on the mean functional image using FSLeyes, with high‐intensity voxels visually confirmed using the corresponding MPRAGE structural image (Han et al., 2023). CSF time series were extracted from both bandpass‐filtered (approximately 0.007–0.1 Hz) and non‐bandpass‐filtered, voxelwise *z*‐score‐normalized data (Figure 2b).

### 2.5 Cross‐correlation function analysis

**FIGURE 3:**
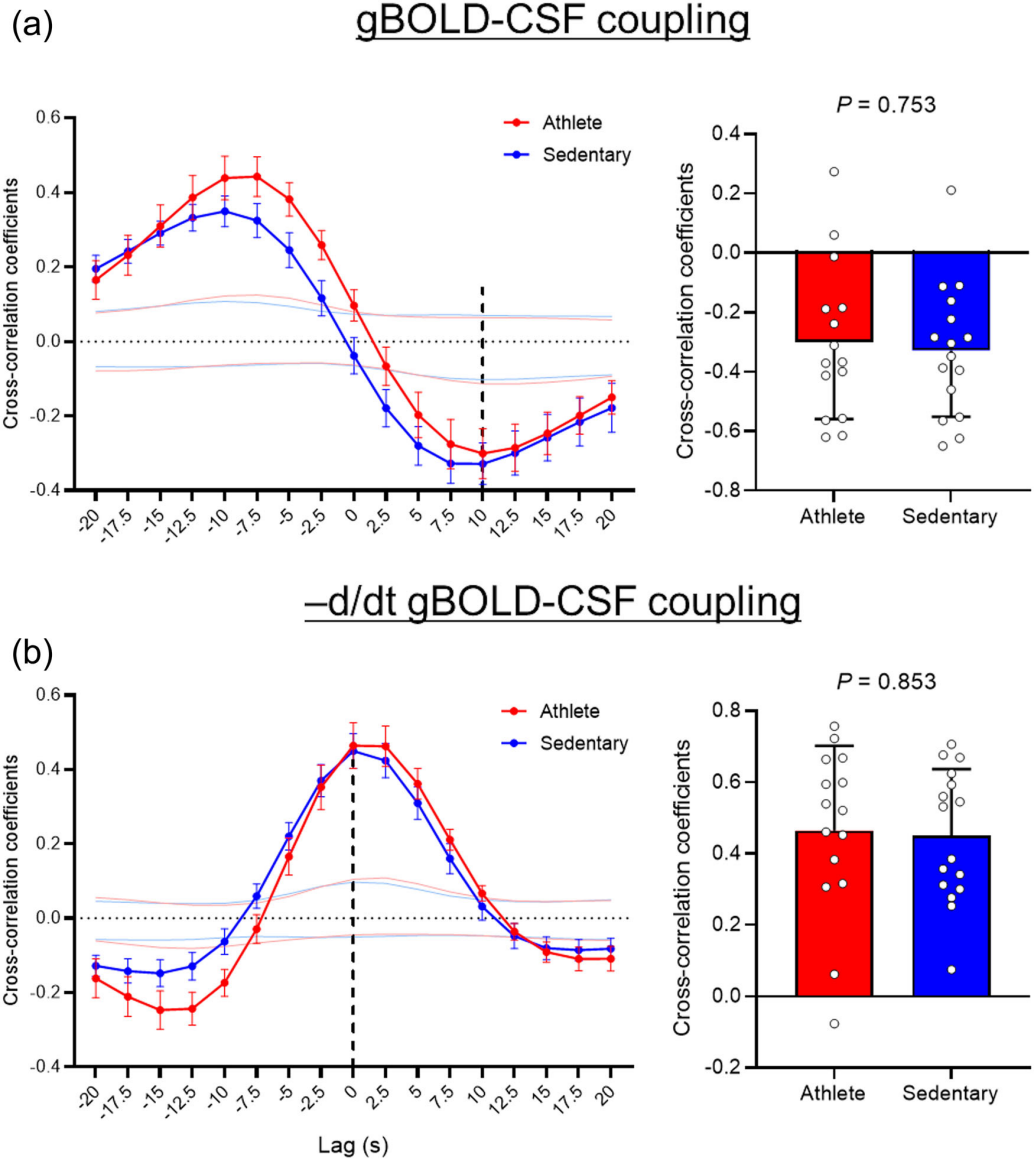
Results of cross‐correlation function analysis showing gBOLD–CSF coupling (a) and time‐derivative gBOLD–CSF coupling (−d/d*t* gBOLD–CSF; b). Data are averaged for the athlete group (*n* = 15, red line and bars) and sedentary group (*n* = 16, blue line and bars). Shaded areas indicate the 95% confidence intervals generated from shuffled signals for each group. Black dashed lines denote the maximum correlation coefficients. Error bars denote standard error (waveforms, left) and standard deviation (bar graphs, right). CSF, cerebrospinal fluid; gBOLD, global blood oxygen level‐dependent.

**FIGURE A1:**
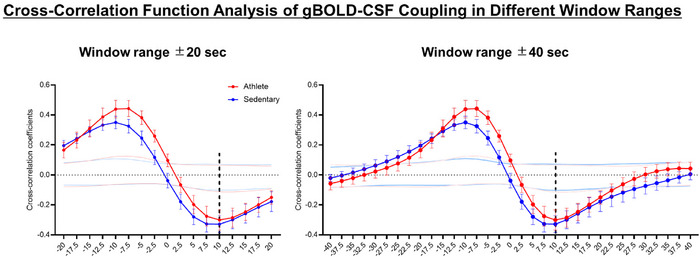
Cross‐correlation function analysis of gBOLD–CSF coupling in different window ranges. To assess the robustness of the cross‐correlation function analysis, we performed a sensitivity analysis using two different window ranges. Cross‐correlation function analyses using two different window ranges yielded identical results, with both analyses showing a peak correlation at the same lag (+10 s). No significant correlations were observed outside the ±20 s range. Specifically, in the athlete group, correlation coefficients were not significant in the −40 to −27.5 s and 25 to 40 s ranges (*P* > 0.05), while in the sedentary group, no significant correlations were detected in the −40 to −30 s and 30 to 40 s ranges (*P* > 0.05).

We performed cross‐correlation function analysis (Appendix Table A2), which has been widely used in previous gBOLD–CSF coupling studies (Bolt et al., 2025; Fultz et al., 2019; Han et al., 2021; Han, Lee et al., 2024). Consistent with previous reports, CSF signals showed an anticorrelation with gBOLD fluctuations, with a negative correlation peak at a lag of +10 s and a positive peak at –10 s (Figure 3, top). The negative peak at +10 s was used as an index of gBOLD–CSF coupling, reflecting the delayed CSF inflow response following global brain‐wide decreases in blood volume/oxygenation, in line with the Monro–Kellie hypothesis. The cross‐correlation function analysis was also subjected to a sensitivity analysis using different windows (Appendix Figure A1).

In addition, we calculated time‐derivative gBOLD–CSF coupling (−d/d*t* gBOLD–CSF) by taking the temporal derivative of the gBOLD signal, multiplying by −1, and rectifying it (i.e., setting negative values to zero; Figure 3b). This transformation emphasizes rapid downward transitions in gBOLD, which are thought to correspond to synchronized reductions in intracranial blood volume. The −d/d*t* gBOLD–CSF coupling showed its strongest correlation at zero lag, highlighting the near‐synchronous relationship between sharp gBOLD decreases and CSF inflow. This derivative‐based index therefore provides complementary information by capturing the more immediate component of gBOLD–CSF coupling, whereas the standard gBOLD–CSF index primarily reflects the delayed component.

Statistical significance of coupling was assessed using permutation testing. Session IDs of the gBOLD signals were randomly shuffled, and cross‐correlation functions were recalculated with the CSF signals. This procedure was repeated 10,000 times to generate a null distribution. *P‐*values were derived by comparing the observed coupling strengths (the +10 s peak for gBOLD–CSF coupling and the 0 s peak for −d/d*t* gBOLD–CSF coupling) against this null distribution.

### 2.6 Spectral and transfer function analyses

Non‐bandpassed gBOLD and CSF signals were used for spectral and transfer function analyses to evaluate frequency dependence and amplification (gain) not captured by cross‐correlation function. Spectral and transfer function analysis enables the characterization of the dynamic relationship between two signals within the frequency domain, allowing the calculation of signal power, coherence, gain and phase (Zhang et al., 1998). Following earlier methods (Qin et al., 2024; Zhu et al., 2015), time series were linearly interpolated, resampled at 1 Hz, and detrended by third‐order polynomial fitting. Data were subdivided into 100‐s segments with 50% overlap and Hanning windowing, giving a 0.01 Hz spectral resolution. Power spectral density quantified signal fluctuation magnitude, while transfer function analysis characterized the frequency‐dependent gBOLD–CSF coupling with gain, phase, and coherence. Gain quantified the magnitude relation between gBOLD and CSF signals, while phase indicated their temporal relation. Coherence ranges from 0 to 1, with higher values indicating a stronger linear relationship at that frequency (a frequency‐domain analogue of variance explained). Power spectral density and transfer function parameters were calculated in very low‐frequency (VLF, 0.01–0.03 Hz) and low‐frequency (LF, 0.03–0.07 Hz) bands, reflecting vasomotor waves with distinct peaks around 0.03 Hz (Kiviniemi et al., 2016). The slower component (<0.03 Hz) may reflect combined sympathetic and parasympathetic influences, while the faster component (0.03–0.07 Hz) is considered predominantly parasympathetic (Akselrod et al., 1981; Biswal et al., 1995; Kiviniemi, 2008). Frequency‐domain analyses were performed using DADiSP (DSP Development Corporation, Newton, MA, USA).

### 2.7 Physical activity and ${\dot{V}}_{O_{2}\max}$

Weekly physical activity was estimated using a modified Godin Leisure‐Time Exercise Questionnaire (Ainsworth et al., 2011) and expressed as metabolic equivalents (METs). ${\dot{V}}_{O_{2}\max}$, an objective measure of cardiorespiratory fitness, was obtained in athletes using treadmill testing (ORK‐7000; Ohtaki Root Kogyo, Iwate, Japan) with a breath‐by‐breath gas analyser (AE‐310S, Minato Medical Science, Kobe, Japan). ${\dot{V}}_{O_{2}}$ was averaged and output every 15 s, and ${\dot{V}}_{O_{2}\max}$ was determine as the maximum value over the final 1 min of test. ${\dot{V}}_{O_{2}\max}$ was confirmed if three of the following criteria were met (Takayama et al., 2017): (1) a levelling off in ${\dot{V}}_{O_{2}}$ despite an increase in a treadmill velocity; (2) respiratory quotient ≥1.1; (3) heart rate ≥90% of age‐predicted maximum; (4) Borg scale ≥19 (Borg, 1990). A prior study in this cohort demonstrated cardiac adaptations typically observed in endurance athletes (Fukuie et al., 2024).

### 2.8 Sample size estimate

Sample size was estimated using G*Power (v3.1, Heinrich Heine Universität Düsseldorf, Düsseldorf, Germany) for a between‐group comparison of gBOLD–CSF coupling. Because no prior studies have directly examined gBOLD–CSF coupling in athletes, we referred to earlier findings that athletes showed higher vagal activity assessed by heart rate variability when compared with sedentary individuals (effect size = 1.03; two‐sided α = 0.05; power = 0.8; groups = 2) (Dixon et al., 1992). Although heart rate variability is not a direct measure of gBOLD–CSF coupling, it may reflect differences in the autonomic nervous activity relevant to the outcome. Based on this effect size, a sample size of 16 participants per group was estimated to provide 80% statistical power to detect between‐group differences.

### 2.9 Statistical analysis

Normality of data distributions was tested using the Shapiro–Wilk test, supplemented with visual inspections of histograms and Q–Q plots. Depending on normality, athlete and sedentary groups were compared using independent Student's *t*‐test or the Mann‐Whitney *U*‐test. Group, frequency band, and their interaction effects on power spectral density and transfer function metrics were analysed using linear mixed models. The fixed effects of the model were specified as β_0_ + β_1_Group + β_2_Frequency Band + β_3_(Group × Frequency Band). The random‐effects structure included a subject‐specific random intercept (1∣Subject). In the present study, the sample size was relatively small, and frequency band was treated as a within‐subject factor without temporal continuity; therefore, overly complex covariance structures were avoided. Accordingly, a diagonal covariance structure, assuming independence among within‐subject measurements, was adopted in the linear mixed‐effects model. When compared with representative covariance structures such as unstructured and compound symmetry, their structure resulted in convergence warning or the diagonal structure yielded the lowest Akaike information criterion score. Models were estimated using restricted maximum likelihood, and degrees of freedom were calculated using the Satterthwaite approximation. *Post hoc* pairwise comparisons were conducted using estimated marginal means with Bonferroni correction. Model assumptions were assessed based on convergence diagnostics and inspection of residual distributions. Statistical significance was defined as *P *< 0.05. Results are presented as means ± standard deviations, 95% confidence intervals, or standard errors as appropriate. Analyses were performed using SPSS version 25 (IBM Corp., Armonk, NY, USA) and MATLAB (R2024a, MathWorks, Inc., Natick, MA, USA).

## 3 RESULTS

### 3.1 Participant characteristics

Table 1 summarizes the characteristics of the athlete and sedentary groups. The two groups were similar in height, BMI, and systolic blood pressure. Body mass was significantly lower, and weekly METs were significantly higher in the athlete group compared with the sedentary group. Average ${\dot{V}}_{O_{2}\max}$ in the athlete group was 69.5 mL/kg/min, which corresponds to >90th percentile for the age‐ and sex‐matched general population (Graves et al., 2015). Resting heart rate and diastolic blood pressure were significantly lower in the athlete group.

**TABLE 1:** Characteristics of the endurance athlete and sedentary groups.

	Athlete (*n* = 15)	Sedentary (*n* = 16)	*P*
Age (years)	20 ± 1	21 ± 2	0.264
Height (cm)	171 ± 6	172 ± 6	0.545
Body mass (kg)	58 ± 6	66 ± 12	**0.026**
Body mass index (kg/m^2^)	20 ± 1	22 ± 4	0.151
Weekly METs (3.5 mL/kg/min)	108 ± 22	13 ± 8	**<0.001**
${\dot{V}}_{O_{2}\max}$ (mL/kg/min)	69.5 ± 3.1	—	
Heart rate (bpm)	53 ± 6	67 ± 13	**0.001**
Systolic blood pressure (mmHg)	114 ± 10	115 ± 8	0.117
Diastolic blood pressure (mmHg)	63 ± 8	67 ± 7	**0.031**

Data are shown by means ± standard deviation (SD). *P*‐values < 0.05 are bold. ${\dot{V}}_{O_{2}\max}$ was measured only in athletes. Some of the data in this table have been reported in our previous studies. METs, metabolic equivalent; ${\dot{V}}_{O_{2}\max}$, maximal oxygen uptake.

### 3.2 Cross‐correlation function analysis

Figure 3 illustrates the results of the cross‐correlation function analysis. Consistent with prior studies examining gBOLD–CSF coupling with cross‐correlation function analysis (Fultz et al., 2019; Han et al., 2021), both groups showed the biphasic gBOLD–CSF cross‐correlation pattern, with the strongest negative correlation at a lag of +10 s (athletes: *r* = −0.301 ± 0.259, *P* < 0.001, 10,000 permutations vs. sedentary: *r* = −0.328 ± 0.224, *P* < 0.001, 10,000 permutations). Similar results were obtained in the sensitivity analysis using different windows (Figure 1a). For the −d/d*t* gBOLD–CSF coupling, both groups showed the strongest positive correlation at zero lag (athletes: *r* = 0.464 ± 0.237, *P* < 0.001, 10,000 permutations vs. sedentary: *r* = 0.450 ± 0.186, *P* < 0.001, 10,000 permutations). No significant between‐group differences were observed for either gBOLD–CSF (*P* = 0.753) or −d/d*t* gBOLD–CSF (*P* = 0.853) coupling strengths (Figure 3 and Appendix Table A3).

### 3.3 Spectral analysis

**FIGURE 4:**
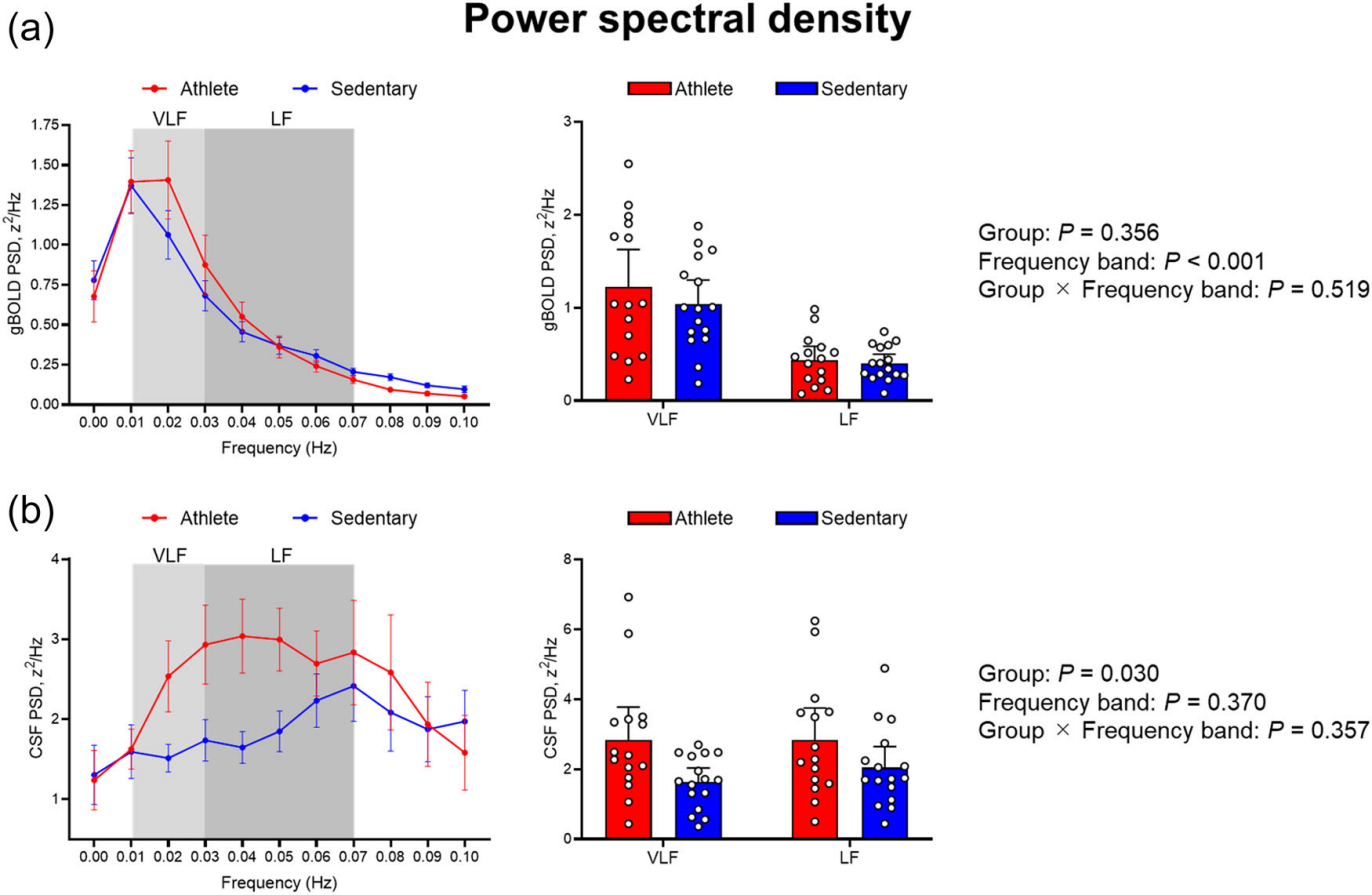
Results of spectral analysis showing the power spectral density waveforms of gBOLD (a) and CSF inflow signals (b) within the 0.01–0.1 Hz range. Power spectral density from the very low‐frequency (VLF: 0.01–0.03 Hz) and low‐frequency (LF: 0.03–0.07 Hz) bands was compared between the athlete group (*n* = 15, red line and bars) and the sedentary group (*n* = 16, blue line and bars). Data are shown as means with standard errors (waveforms, left) and as group means with 95% confidence intervals (bar graphs, right). White circles denote individual data points. Group, frequency band, and group × frequency band effects were assessed using a linear mixed model with Bonferroni correction for *post hoc* comparisons. CSF, cerebrospinal fluid; gBOLD, global blood oxygen level‐dependent.

Figure 4 and Appendix Table A4 present the power spectral density of the gBOLD and CSF signals. For the gBOLD, no significant effects of group or group × frequency band interaction were found (VLF: 1.23 [–9.24, 11.69] *z* ^2^/Hz and LF: 0.44 [−1.16, 2.04] *z* ^2^/Hz in athlete group vs. VLF: 1.04 [−0.99, 3.07] *z* ^2^/Hz and LF: 0.40 [−1.25, 2.06] *z* ^2^/Hz in sedentary group). But, a significant effect of frequency band was observed, with higher power in the VLF band (1.13 [−0.63, 2.90] *z* ^2^/Hz) compared with the LF band (0.42 [−1.15, 1.99] *z* ^2^/Hz) across both groups.

In contrast, power spectral density of the CSF inflow signal showed a significant group effect across both VLF and LF bands, with higher power in the athlete group than in the sedentary group (2.83 [2.19, 3.47] *z* ^2^/Hz in athlete group vs. 1.84 [1.22, 2.46] *z* ^2^/Hz in sedentary group). No significant effects of frequency band or group × frequency band interaction were found (VLF: 2.84 [2.15, 3.52] *z* ^2^/Hz and LF: 2.83 [2.08, 3.58] *z* ^2^/Hz in athlete group vs. VLF: 1.63 [0.96, 2.30] *z* ^2^/Hz and LF: 2.05 [1.32, 2.77] *z* ^2^/Hz in sedentary group).

### 3.4 Transfer function analysis

**FIGURE 5:**
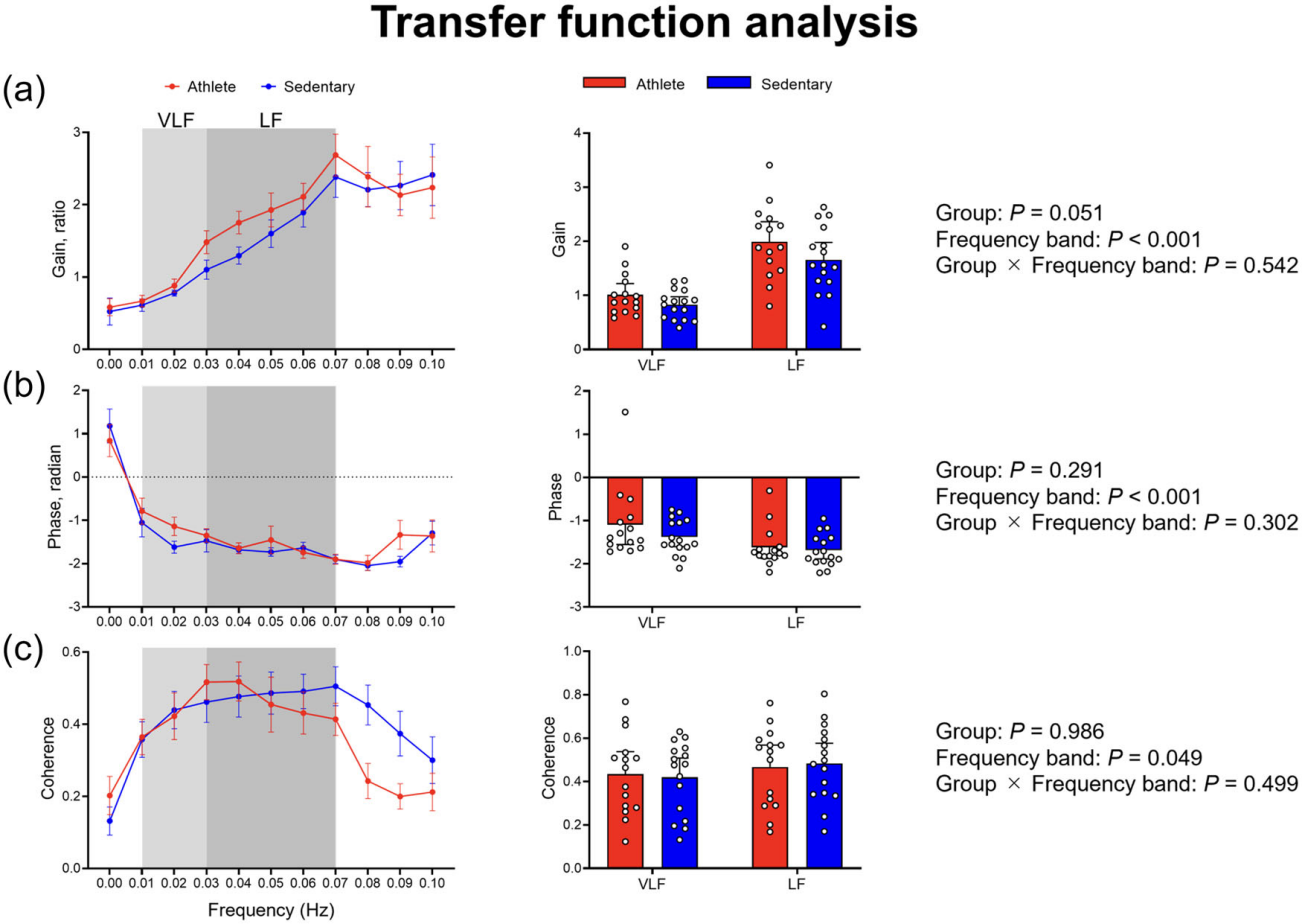
Results of transfer function analysis showing gain (a), phase (b), and coherence (c) between gBOLD and CSF inflow signals within the 0.01–0.1 Hz range. Transfer function metrics from the very low‐frequency (VLF: 0.01–0.03 Hz) and low‐frequency (LF: 0.03–0.07 Hz) bands were compared between the athlete group (*n* = 15, red line and bars) and the sedentary group (*n* = 16, blue line and bars). Data are shown as means with standard errors (waveforms, left) and as group means with 95% confidence intervals (bar graphs, right). White circles denote individual data points. Group, frequency band, and group × frequency band effects were assessed using a linear mixed model with Bonferroni correction for *post hoc* comparisons. CSF, cerebrospinal fluid; gBOLD, global blood oxygen level‐dependent.

Figure 5 and Appendix Table A5 show transfer function gain, phase and coherence between gBOLD and CSF signals. Although the group effect did not reach statistical significance (athlete vs. sedentary: 1.50 [−0.36 3.36] vs. 1.24 [−0.06 2.55]; *P* = 0.051), moderate effect size was observed (Cohen's *d* = 0.523). Furthermore, the group × frequency band interaction was not significant (VLF: 1.01 [−0.83, 2.85] and LF: 1.99 [−0.03, 4.02] in athletes; VLF: 0.83 [−0.33, 1.99] and LF: 1.65 [−0.35, 3.66] in sedentary participants). No significant effects of group or group × frequency band interaction were found for phase (VLF: −1.09 [−1.44, −0.75] radians and LF: −1.62 [−1.84, −1.40] radians in athlete group vs. VLF: −1.38 [−1.71, −1.05] radians and LF: −1.69 [−1.90, −1.47] radians in sedentary group) or coherence (VLF: 0.44 [0.34, 0.53] and LF: 0.47 [0.37, 0.56] in athlete group vs. VLF: 0.42 [0.33, 0.51] and LF: 0.48 [0.39, 0.58] in sedentary group).

A significant effect of frequency band was observed for all transfer function metrics (*P* < 0.05). Specifically, coherence and gain were higher in the LF band (coherence: 0.48 [0.41, 0.54]; gain: 1.82 [0.23, 3.42]) compared with the VLF band (coherence: 0.43 [0.36, 0.49]; gain: 0.92 [0.03, 1.81]). Phase was lower in the LF band (−1.65 [−1.81, −1.50] radians) compared with the VLF band (−1.24 [−1.48, −1.00] radians).

## 4 DISCUSSION

This study compared gBOLD–CSF coupling between young male endurance athletes and age‐ and sex‐matched sedentary individuals using cross‐correlation and transfer function analyses. The main findings were as follows. First, cross‐correlation‐derived gBOLD–CSF coupling did not differ significantly between groups. Second, spectral analysis showed that CSF power was significantly higher in athletes. Finally, significant frequency effects were found for gBOLD power as well as for transfer function gain, phase and coherence across groups. Taken together, these results indicate that endurance training may be associated with greater CSF fluctuations at rest while not affecting gBOLD–CSF coupling. Also, gBOLD–CSF coupling seems to be modulated by frequency‐dependent physiological processes that could involve autonomic nervous regulation.

In this study, we hypothesized that athletes would exhibit larger CSF signal fluctuations accompanied by greater fluctuations in the gBOLD signal, resulting in stronger gBOLD–CSF coupling compared with sedentary individuals. However, gBOLD fluctuations did not differ significantly between the two groups, and coupling strength based on cross‐correlation analysis was comparable between athletes and sedentary participants. One possible explanation is that gBOLD–CSF coupling may not be substantially altered by endurance training in young healthy adults. A large cross‐sectional study of healthy adults aged 36–100 years showed that cross‐correlation‐derived coupling remains relatively stable until midlife, after which it declines with advancing age (Han, Liu et al., 2024). Thus, age rather than endurance training may play a more prominent role in determining cross‐correlation‐derived coupling in healthy individuals. Another possibility is that cross‐correlation function analysis has limited sensitivity to detect exercise‐related changes. Because cross‐correlation function compares bandpassed gBOLD and CSF signals within a restricted frequency range (0.01–0.1 Hz) and at a single time lag, frequency‐specific differences may not be captured. Recognizing this limitation, we considered spectral and transfer function analyses as supplementary approaches to provide complementary information.

Power spectral density analysis indicated that CSF power was higher in athletes, whereas gBOLD power did not differ between groups. In addition, transfer function gain, representing the amplification ratio of CSF relative to gBOLD, did not show a statistically significant group difference (*P* = 0.051), although a moderate effect size was observed (Cohen's *d* = 0.523). These findings suggest that athletes have larger CSF fluctuations relative to gBOLD fluctuations, although the physiological implications of this observation remain to be clarified. The interpretation is broadly consistent with findings from a recent trial in which a 12‐week cycling intervention increased CSF flow dynamics and altered meningeal lymphatic vessels as measured with contrast‐enhanced MRI (Yoo et al., 2025). Although the methodologies differ, this previous and our studies raise the possibility that aerobic exercise could influence CSF dynamics in healthy adults (Tarumi, Yamabe, Fukuie, Zhu et al., 2021).

Because gBOLD power was comparable across groups, the elevated CSF power in athletes is unlikely to be explained solely by infra‐slow global brain BOLD activity. Although the mechanism cannot be elucidated based on the data from the present study, one possible explanation is that factors related to autonomic nervous activity may contribute to this difference. CSF fluctuations have been shown to increase during non‐REM sleep, a parasympathetic‐dominant state (Fultz et al., 2019), along with an expansion of the interstitial space into which CSF flows (Fultz et al., 2019; Xie et al., 2013). Also, endurance training has been suggested to increase parasympathetic activity at rest (Aubert et al., 2003), as shown by lower heart rate in athletes than sedentary individuals in the current study. Taken together, accumulating evidence points to a potential relationship between autonomic nervous activity and CSF flow, and autonomic adaptations in athletes may partially coincide with the greater CSF fluctuations observed in this study. However, in the absence of autonomic measurements at rest and during MRI scanning, no causal inferences can be made, and further investigation is required.

Both gBOLD power and transfer function metrics exhibited frequency dependence. Specifically, power spectral density of gBOLD and phase were higher in the VLF band than in the LF band, whereas coherence and gain were higher in the LF band. The LF band has been associated with parasympathetic‐weighted autonomic influences and tends to show spatially uniform fluctuations across the brain, which may support global clearance processes (Kiviniemi et al., 2016). By contrast, the VLF band likely reflects contributions from both sympathetic and parasympathetic activity and may relate more closely to local cerebral circulation and CSF convection (Kiviniemi et al., 2016). These slow fluctuations are generally attributed to systemic physiological processes such as autonomic nervous modulation and respiration rather than direct neurovascular coupling to neuronal activity. Indeed, sympathetic (Chang et al., 2013; Shmueli et al., 2007) and parasympathetic (Napadow et al., 2008) components of heart rate and respiratory variability (Birn et al., 2006; Chang & Glover, 2009) have been linked to gBOLD fluctuations in the 0.01–0.1 Hz range. As this study did not assess systemic physiological measures, future investigations that integrate gBOLD, CSF dynamics and cardiorespiratory variables will be valuable for clarifying these relationships.

Cross‐correlation function analysis indicated maximal correlation coefficients (absolute *r* ≈ 0.3–0.4) at +10 s, suggesting that CSF signal changes lagged behind gBOLD by about 10 s. In comparison, transfer function phase indicated delays of −0.92 to −2.02 radians (−14.7 to −2.1 s; mean –6.28 ± 3.91 s) within the 0.01–0.1 Hz range. Although we did not directly compare these values, phase at ∼0.02 Hz (−1.39 radian; −11.05 s) showed the closest correspondence with the lag observed in cross‐correlation function, implying that the cross‐correlation‐derived maximum correlation may largely reflect this frequency. Transfer function coherence was ∼0.3 across the 0.01–0.1 Hz range, suggesting that gBOLD fluctuations accounted for about 30% of CSF variance at these frequencies under linear and stationary assumptions. This also indicates that other physiological factors, such as autonomic, cardiac and respiratory influences, likely contribute to CSF dynamics.

### 4.1 Limitations and strengths

This study has several limitations. First, the sample size was small and limited to young men. Therefore, the generalizability of the findings is constrained, and future studies with larger samples that include both sexes and older athletes are warranted. Second, because ${\dot{V}}_{O_{2}\max}$ was measured only in the athlete group, it remains unclear to what extent individual cardiorespiratory fitness, as opposed to endurance training habits, contributed to the observed differences in CSF flow dynamics. Although self‐reported physical activity was assessed in both groups and substantial differences were evident, the lack of objective fitness data in sedentary participants limits direct group comparability. Moreover, we cannot exclude the possibility that some athletes were naturally fitter prior to engaging in long‐term endurance training. Future studies incorporating objective fitness assessments in all participants will be essential to disentangle the relative contributions of fitness and training exposure. Third, gBOLD–CSF coupling is an indirect index and not a direct measure of glymphatic function. Although previous studies have linked this coupling to ageing and Alzheimer's disease pathology (Han et al., 2021, 2023; Han, Lee et al., 2024, 2024), the interpretation of CSF fluctuations as clearance efficiency should remain cautious. Direct measurement of cerebral metabolic rate (e.g., PET, SPECT) would help clarify the true relationship between exercise and brain's metabolic waste clearance. Fourth, the cross‐sectional design precludes causal inference regarding the effects of endurance training on CSF dynamics. Fifth, although athletes were instructed to avoid high‐intensity training for 24 h prior to MRI scanning, the timing and intensity of the most recent exercise session, as well as sleep quality on the preceding night, were not assessed. These factors may have influenced our results and should be controlled for in future studies. Lastly, the physiological interpretation of VLF and LF bands remains tentative, as these fluctuations may reflect multiple systemic processes including autonomic nervous regulation and respiration (Akselrod et al., 1981; Chang et al., 2013; Kiviniemi et al., 2016). Future studies with larger, sex‐diverse samples, longitudinal or interventional designs, and multimodal physiological measurements will be needed to confirm and extend the present findings.

Despite these limitations, the study has strengths. To our knowledge, this is the first investigation of gBOLD–CSF coupling in endurance athletes, a group characterized by high‐intensity aerobic training and associated cardiovascular and structural adaptations (Fukuie et al., 2024; Tarumi et al., 2022; Tarumi, Yamabe, Fukuie, Kimura et al., 2021). The combined use of cross‐correlation function and transfer function analyses provided complementary insights into both overall and frequency‐dependent coupling mechanisms, which may not be fully captured by cross‐correlation function alone.

### 4.2 Future directions

Previous studies have reported that endurance athletes exhibit enhanced resting parasympathetic activity as a result of training (Aubert et al., 2003), and consistent with this, resting heart rate was lower in the endurance athletes than in the sedentary control group in the present study. However, because the present study is a cross‐sectional design and did not assess autonomic nervous activity (e.g., heart rate variability) during MRI measurement, the relationship between endurance training‐induced changes in autonomic activity and brain waste clearance remains unclear. Notably, CSF dynamics are closely linked to autonomic activity (Bolt et al., 2025), and prior studies have shown that CSF dynamics are attenuated in response to acute stimuli associated with sympathetic activation such as exercise (Tarumi, Yamabe, Fukuie, Zhu et al., 2021) and visual stimulation (Williams et al., 2023). These findings collectively suggest that a reduction in sympathetic activity or an increase in parasympathetic activity may be associated with enhanced fluctuations in CSF flow. Importantly, aerobic exercise training interventions have been shown to increase parasympathetic activity (Grässler et al., 2021) in non‐athletic adults, indicating that these autonomic adaptations are not limited to trained athletes. Therefore, future studies adopting an interventional design and incorporating measurements of autonomic activity are needed to clarify the effects of endurance training on CSF‐mediated brain waste clearance.

### 4.3 Conclusions

Endurance athletes exhibited gBOLD–CSF coupling comparable to that of sedentary individuals in the time domain, as assessed by cross‐correlation analysis. In contrast, frequency‐domain analyses revealed greater CSF power in athletes despite no corresponding group differences in gBOLD power. In both groups, transfer function gain, phase and coherence between gBOLD and CSF signals demonstrated clear frequency‐dependent patterns across very low‐ and low‐frequency bands. Collectively, these findings suggest that endurance training may be associated with altered CSF dynamics, characterized primarily by increased amplitude of slow CSF fluctuations, while preserving the temporal coupling between gBOLD and CSF signals.

## AUTHOR CONTRIBUTIONS

The results of the present study do not constitute endorsement by The Physiological Society and are presented clearly, honestly, and without fabrication, falsification, or inappropriate data manipulation. Takashi Tarumi conceived and designed research; Marina Fukuie and Takashi Tarumi performed experiments; Daisuke Hoshi and Takashi Tarumi analysed data, interpreted results of experiments, prepared figures; Daisuke Hoshi drafted manuscript. All authors have read and approved the final version of this manuscript and agree to be accountable for all aspects of the work in ensuring that questions related to the accuracy or integrity of any part of the work are appropriately investigated and resolved. All persons designated as authors qualify for authorship, and all those who qualify for authorship are listed.

## CONFLICT OF INTEREST

All authors report no disclosures relevant to this manuscript.

## Data Availability

The data that support the findings of this study are available on request from the corresponding author. The data are not publicly available due to privacy or ethical restrictions.

## 1

Functional MRI image processing script is provided below. This script illustrates the image‐processing workflow used in the present study. File paths and filenames are shown as examples and may vary across participants.

COBIDAS compliance (Nichols et al., 2017)

- All preprocessing steps followed COBIDAS recommendations for fMRI reproducibility.
- Software versions, operating system, and parameter choices are reported in the main Methods section.
- The present script is provided for transparency and reproducibility.

**TABLE A1:** Preprocessing steps and codes used to extract gBOLD and CSF signals.

Step	Processing stage	Purpose	Input (file name)	Program	Code / Command
**1**	**Structural MRI data preprocessing**				
1.1	Skull stripping	Generate brain‐extracted structural image	T1‐weighted structural image (MPRAGE.nii.gz)	FreeSurfer	mri_synthstrip ‐i MPRAGE.nii.gz ‐o highres_brain.nii.gz ‐m highres_brain_mask.nii.gz –no‐csf
1.2	Tissue segmentation	Segmentation of GM, WM, and CSF	Brain‐extracted T1 image (highres_brain.nii.gz)	FSL	fast ‐o highres highres_brain.nii.gz
1.3	Mask generation	GM mask creation	GM partial volume map generated from Step 1.2 (highres_pve_1.nii.gz)	FSL	fslmaths highres_pve_1.nii.gz ‐thr 0.5 ‐bin gray_mask.nii.gz
**2**	**Rs‐fMRI data preprocessing**				
2.1	Preprocessing for gBOLD	Slice‐timing correction, removal of first 10 volumes, motion correction, skull stripping, and high‐pass filtering. Generation of a matrix file for structural‐to‐functional registration (used for gBOLD analyses)	Raw rs‐fMRI data and brain‐extracted T1 image (highres_brain.nii.gz)	FSL	FEAT (GUI‐based)
2.2	Preprocessing for CSF	Slice‐timing correction and removal of first 10 volumes (motion correction and high‐pass filtering not applied)	Raw rs‐fMRI data	FSL	FEAT (GUI‐based)
2.3	CSF mask generation	Define CSF's ROI	MPRAGE and mean functional image	FSL	Manual delineation using FSLeyes (mask: CSF_mask_bottom_slice.nii.gz)
**3**	**Registration of structural mask to functional space**				
3.1	Linear registration	Align grey matter mask to functional space	GM mask generated from Step 1.3 (gray_mask.nii.gz) and example functional image (example_func.nii.gz) and matrix file (highres2example_func.mat) from FEAT in Step 2.1	FSL	flirt ‐in gray_mask.nii.gz ‐ref example_func.nii.gz ‐applyxfm ‐init highres2example_func.mat ‐interp nearestneighbour ‐out gray_mask_func.nii.gz
**4**	**Normalization**				
4.1	Temporal normalization (performed for gBOLD and CSF)	Computing temporal standard deviation and Z‐score normalization	FEAT output files (filtered_func_data.nii.gz and mean_func.nii.gz): gBOLD in Step 2.1 and CSF in Step 2.2	FSL	fslmaths filtered_func_data.nii.gz ‐Tstd filtered_func_data_std.nii.gz; fslmaths filtered_func_data.nii.gz ‐sub mean_func.nii.gz ‐div filtered_func_data_std.nii.gz filtered_func_data_Z.nii.gz
**5**	**Band‐pass (BP) filtering and signal extraction**				
5.1	Temporal filtering (performed for gBOLD and CSF)	Band‐pass filtering at approximately 0.007–0.1 Hz	Preprocessed rs‐fMRI data (filtered_func_data_Z)	FSL	fslmaths filtered_func_data_Z.nii.gz ‐bptf 28.57 2.0 filtered_func_data_Z_bp.nii.gz
5.2	Signal extraction (performed for BP and non‐BP normalized data)	gBOLD signal	BP or non‐BP preprocessed rs‐fMRI data (filtered_func_data_Z_bp.nii.gz or filtered_func_data_Z.nii.gz) and GM mask (gray_mask_func.nii.gz)	FSL	BP: fslmeants ‐i filtered_func_data_Z_bp.nii.gz ‐m gray_mask_func.nii.gz ‐o gBOLD_Z_bp.txt; Non‐BP: fslmeants ‐i filtered_func_data_Z.nii.gz ‐m gray_mask_func.nii.gz ‐o gBOLD_Z.txt
5.3	Signal extraction (performed for BP and non‐BP normalized data)	CSF signal	BP or non‐BP preprocessed rs‐fMRI data (filtered_func_data_Z_bp.nii.gz or filtered_func_data_Z.nii.gz) and CSF mask (CSF_mask_bottom_slice)	FSL	BP: fslmeants ‐i filtered_func_data_Z_bp.nii.gz ‐m CSF_mask_bottom_slice.nii.gz ‐o CSF_Z_bp.txt; Non‐BP: fslmeants ‐i filtered_func_data_Z.nii.gz ‐m CSF_mask_bottom_slice.nii.gz ‐o CSF_Z.txt
**6**	**Brain volume estimation**				
6.1	Structural volume estimation	Brain volume estimation	MPRAGE and FLAIR images (MPRAGE.nii.gz and FLAIR.nii.gz)	FreeSurfer	recon‐all ‐s subj_ID ‐i MPRAGE.nii.gz ‐FLAIR FLAIR.nii.gz ‐all

BP, band‐pass; CSF, cerebrospinal fluid; FLAIR, T2‐weighted fluid‐attenuated inversion recovery; fMRI, functional magnetic resonance imaging; gBOLD, global grey matter blood oxygen level‐dependent; GM, grey matter; GUI, graphical user interface; MPRAGE, T1‐weighted 3D magnetization‐prepared rapid acquisition with gradient echo; ROI, region‐of‐interest; rs‐fMRI, resting‐state functional MRI; WM, white matter.

**TABLE A2:** Codes used for cross‐correlation function analysis.

Step	Analysis stage	Purpose	Program	Script/code
1	Cross‐correlation analysis	Quantify temporal relationship between gBOLD and CSF	MATLAB	n_lag = 21; lags = –10:10;for i = 1:n_sessioncc_real(i,:) = lagged_pearson(x, y, 10); end
2	Permutation testing	Generate null distribution	MATLAB	n_perm = 10000;idx = randperm(n_session);cc_null(p,:) = mean(cc_temp,1);
3	Statistical inference	*P*‐value estimation	MATLAB	p_vals = mean(abs(cc_null) > = abs(mean_real), 1);

**TABLE A3:** Results of cross‐correlation function analysis over lag times from −20 to +20 s showing gBOLD–CSF coupling and time‐derivative gBOLD–CSF coupling in endurance athletes (*n* = 15) and sedentary controls (*n* = 16) (numerical data of Figure 3).

		Lag (sec)																
Group		−20.0	−17.5	−15.0	−12.5	−10.0	−7.5	−5.0	−2.5	0.0	2.5	5.0	7.5	10.0	12.5	15.0	17.5	20.0
gBOLD–CSF coupling																		
Athlete	mean	0.165	0.232	0.310	0.387	0.439	0.443	0.382	0.259	0.097	−0.066	−0.197	−0.275	−0.301	−0.285	−0.246	−0.198	−0.150
	SD	0.201	0.207	0.219	0.230	0.227	0.207	0.174	0.151	0.163	0.199	0.236	0.257	0.259	0.245	0.221	0.194	0.175
Sedentary	mean	0.196	0.242	0.291	0.332	0.350	0.325	0.245	0.116	−0.038	−0.179	−0.280	−0.327	−0.328	−0.299	−0.258	−0.216	−0.178
	SD	0.143	0.128	0.128	0.144	0.166	0.181	0.188	0.190	0.194	0.201	0.207	0.214	0.224	0.237	0.249	0.259	0.263
−d/d*t* gBOLD–CSF coupling																		
Athlete	mean	−0.162	−0.211	−0.247	−0.243	−0.173	−0.029	0.166	0.352	0.464	0.463	0.362	0.211	0.067	−0.036	−0.091	−0.109	−0.109
	SD	0.202	0.208	0.199	0.171	0.141	0.149	0.193	0.230	0.237	0.210	0.161	0.110	0.081	0.087	0.106	0.122	0.128
Sedentary	mean	−0.128	−0.142	−0.148	−0.129	−0.063	0.060	0.220	0.370	0.450	0.424	0.310	0.161	0.032	−0.048	−0.080	−0.086	−0.082
	SD	0.113	0.130	0.145	0.148	0.137	0.130	0.147	0.173	0.186	0.184	0.174	0.161	0.148	0.135	0.123	0.115	0.111

Data are shown as means and standard deviation (SD). CSF, cerebrospinal fluid; gBOLD, global‐blood oxygen level‐dependent.

**TABLE A4:** Results of spectral analysis showing the power spectral density waveforms of gBOLD and CSF inflow signals within the 0–0.1 Hz range in endurance athletes (*n* = 15) and sedentary controls (*n* = 16) (numerical data of Figure 4).

Group		Frequency (Hz)										
		0.00	0.01	0.02	0.03	0.04	0.05	0.06	0.07	0.08	0.09	0.10
gBOLD PSD (*z* ^2^/Hz)												
Athlete	mean	0.68	1.40	1.41	0.87	0.55	0.36	0.24	0.16	0.09	0.07	0.05
	SD	0.62	0.75	0.94	0.72	0.36	0.27	0.14	0.09	0.06	0.05	0.04
Sedentary	mean	0.78	1.37	1.06	0.68	0.46	0.37	0.31	0.21	0.17	0.12	0.10
	SD	0.48	0.70	0.61	0.38	0.25	0.21	0.16	0.09	0.08	0.06	0.08
CSF PSD (*z* ^2^/Hz)												
Athlete	mean	1.24	1.63	2.53	2.93	3.04	3.00	2.70	2.83	2.58	1.94	1.58
	SD	1.44	0.96	1.72	1.91	1.79	1.51	1.57	2.53	2.78	2.03	1.82
Sedentary	mean	1.30	1.59	1.51	1.74	1.65	1.85	2.23	2.42	2.08	1.87	1.97
	SD	1.48	1.34	0.69	1.04	0.79	1.02	1.33	1.76	1.93	1.62	1.55

Data are shown as means ± standard deviation (SD). CSF, cerebrospinal fluid; gBOLD, global‐blood oxygen level‐dependent; PSD, power spectral density.

**TABLE A5:** Results of transfer function analysis showing gain, phase, and coherence between gBOLD and CSF inflow signals within the 0–0.1 Hz range in endurance athletes (*n* = 15) and sedentary controls (*n* = 16) (numerical data of Figure 5).

Group		Frequency (Hz)										
		0.00	0.01	0.02	0.03	0.04	0.05	0.06	0.07	0.08	0.09	0.10
Gain (ratio)												
Athlete	mean	0.58	0.67	0.88	1.48	1.75	1.93	2.11	2.69	2.39	2.13	2.24
	SD	0.45	0.31	0.36	0.61	0.61	0.91	0.72	1.12	1.62	1.11	1.64
Sedentary	mean	0.52	0.61	0.78	1.10	1.30	1.60	1.89	2.38	2.21	2.27	2.41
	SD	0.74	0.34	0.17	0.53	0.47	0.76	0.80	1.12	0.94	1.34	1.70
Phase (radians)												
Athlete	mean	0.84	−0.79	−1.14	−1.35	−1.64	−1.46	−1.74	−1.90	−1.98	−1.33	−1.36
	SD	1.44	1.17	0.82	0.60	0.48	1.24	0.53	0.45	0.68	1.29	1.43
Sedentary	mean	1.18	−1.05	−1.62	−1.47	−1.68	−1.73	−1.64	−1.90	−2.05	−1.96	−1.29
	SD	1.57	1.32	0.55	1.03	0.33	0.38	0.52	0.38	0.43	0.49	1.10
Coherence												
Athlete	mean	0.20	0.36	0.42	0.52	0.52	0.45	0.43	0.41	0.24	0.20	0.21
	SD	0.20	0.19	0.25	0.19	0.21	0.29	0.23	0.17	0.19	0.14	0.20
Sedentary	mean	0.13	0.36	0.44	0.46	0.48	0.49	0.49	0.51	0.45	0.37	0.30
	SD	0.16	0.20	0.21	0.23	0.23	0.23	0.19	0.22	0.22	0.25	0.26

Data are shown as means and standard deviation (SD). CSF, cerebrospinal fluid; gBOLD, global‐blood oxygen level‐dependent; PSD, power spectral density.
